# Obstetric management for pregnant women with Klippel–Trenaunay syndrome: A UK case report and review of the literature

**DOI:** 10.1002/ijgo.15889

**Published:** 2024-09-03

**Authors:** Inês F. Silva Correia, Munawar Hussain, Jo‐Anne Johnson

**Affiliations:** ^1^ School of Medicine, Faculty of HEMS Anglia Ruskin University Chelmsford UK; ^2^ Department of Obstetrics and Gynaecology Southend University Hospital Southend‐on‐Sea UK

**Keywords:** c‐section, Klippel–Trenaunay syndrome, obstetrics, pregnancy, vascular malformations

## Abstract

Klippel–Trenaunay Syndrome (KTS) is a rare congenital vascular disorder characterized by extensive capillary and venous malformations that pose unique challenges during pregnancy. This case report discusses the successful management of a 34‐year‐old pregnant woman with KTS who had two caesarean sections, resulting in the birth of two healthy babies. Despite the lack of evidence‐based guidelines for obstetrical management in KTS, a multidisciplinary team collaborated to devise a high‐risk thrombosis management plan, involving the use of compression stocking and low molecular weight heparin prophylaxis. The patient's elevated risk of thrombosis, exacerbated during pregnancy, informed the decision of caesarean sections, aligning with finding that in most KTS pregnancies, this method of delivery based on obstetric indications and arteriovenous malformations is chosen. This case highlights the importance of systematic and patient‐centered care, advocating for comprehensive obstetric management guidelines to address the unique challenges posed by KTS during pregnancy. Further research is warranted to enhance our understanding and refine guidelines for individuals with vascular abnormalities linked to KTS.

## Synopsis

This report discusses the successful management of pregnancy and labor in a woman with Klippel–Trenaunay syndrome, highlighting the importance of multidisciplinary care and thrombosis prophylaxis.

## 1 INTRODUCTION

Klippel–Trenaunay syndrome (KTS) is a rare congenital vascular disorder that typically manifests during childhood. In 30%–60% of cases, it is characterized by a triad of extensive capillary and venous malformations, bone and soft tissue overgrowth, and lymphatic malformations.^1^, ^2^ This rare hematological condition can affect individuals of any racial background or gender and can exhibit varying degrees of severity and phenotypic diversity.^3^ In pregnancy, KTS is associated with an elevated risk of thromboembolic events, significant hemorrhages, and additional vascular complications, particularly heightened in the pre‐partum period but also possible during and after childbirth. Additionally, throughout pregnancy and the early postpartum phase, KTS‐related symptoms can worsen, increasing the risk of pulmonary embolism (PE) and deep vein thrombosis (DVT) by 2.3% and 5.8%, respectively, alongside bleeding from varicose veins.^2^, ^6^, ^7^, ^8^ Typically, the diagnosis of KTS relies on a comprehensive clinical evaluation, including medical history and examination. Imaging studies, such as ultrasound, magnetic resonance imaging (MRI) or angiography, are essential in assessing the extent of vascular malformations, particularly arteriovenous malformations (AVMs). Although this occasionally renders pregnancy unadvisable, it is recognized that with careful monitoring, successful pregnancies can be achieved.^4^, ^5^ The lack of evidence‐based guidelines for the obstetrical management of pregnant women with KTS introduces significant uncertainties, especially regarding pregnancy safety and the selection of appropriate delivery methods to mitigate the risk of thromboembolic or hemorrhagic events. In this report, we discuss the case of a pregnant woman with KTS who underwent two successful caesarean sections, resulting in the birth of two healthy babies, without any vascular complications.

## 2 CASE REPORT

We present a case of a 34‐year‐old multiparous woman diagnosed with KTS who underwent two uncomplicated caesarean sections at a university hospital in the UK. For this case, ethical approval and patient consent have been sought. The diagnosis of KTS at the age of 8 years was determined by the observation of a port wine stain affecting the lateral three toes of her left foot, alongside significant venous dilation in her left lower leg. Moreover, during an assessment at 18 years of age by a vascular surgeon, it was noted that her left leg exceeded her right leg in length by 2.5 cm. The patient described experiencing sporadic painful episodes in the regions of these malformations, intensified by applied pressure. No further documentation was available that described the patient's symptoms between this age and her first pregnancy journey at the university hospital.

The patient has no history of thrombotic events but chooses to take the combined oral contraceptive pill. Consequently, her general practitioner monitors her closely to assess for any exacerbation of KTS. The patient's father has a history of heart disease and hypertension, attributed to lifestyle factors, and her paternal grandfather has type 2 diabetes mellitus. There is no family history of clotting‐related diseases.

During both pregnancies, at ages 32 and 34 years, a physical examination revealed an enlargement and elongation of the patient's left leg compared to the right leg, accompanied by visible venous dilation and swelling. In addition, a small non‐tender port wine stain was identified on the lateral three toes of the left leg foot, resembling the one identified in childhood. These signs are frequently associated with an elevated risk of intravascular coagulopathy in KTS leading to thrombotic events and pain in the affected limb. AVMs were not identified in our patient on screening.

The patient's high‐risk thrombosis management plan remained consistent during both pregnancies and postnatal periods. This plan involved continuous compression stocking alongside antenatal prophylaxis with 40 mg low molecular weight heparin (LMWH) injections starting at 28 weeks of gestation. These injections were temporarily ceased for a minimum of 36 hours before labor and resumed promptly after birth for a postpartum period of 6 weeks. However, during the patient's first pregnancy, in 2021, injections were initiated at 19 weeks in response to mild joint pain in the left knee and ankle. She was also advised to take paracetamol as needed for pain management.

Blood tests were performed monthly following the start of injections, transitioning to biweekly assessments from 36 weeks of gestation to monitor hemoglobin and platelet levels, alongside coagulation function. Despite revealing a decrease in platelet levels in her first pregnancy, the findings did not warrant discontinuing the LMWH injections. Additionally, antenatal clinic follow‐ups were arranged in both pregnancies to monitor for any limb swelling, vaginal discharge, or variceal bleeds.

In her first pregnancy, at 37 + 6 weeks gestation, the patient reported a vulvovaginal lump, and at 38 + 3 weeks, she experienced a substantial increase in left leg vein swelling. Once an MRI ruled out an AVM and a DVT was ruled out in the leg, the patient was advised to monitor the progression of the swelling and seek medical attention if excessive bleeding from the vaginal varicose vein occurred.

Despite the patient's initial preference for a natural birth, due to heightened thrombotic risk, slow labor progression despite oxytocin administration, and fetal distress, a category 2 caesarean section was considered a safer alternative for her first pregnancy. This decision was followed by an extensive multidisciplinary team (MDT) consultation involving anesthesiology, hematology, and obstetrics, considering the patient's clinical condition and gestational age. In her most recent pregnancy, the birth of a healthy 3.145‐kg baby was also successfully achieved via a planned category 3 caesarean section. Interestingly, both deliveries occurred at the same gestational age of 38 + 4 weeks, with no complications reported during or after childbirth. Both infants are thriving and healthy without any postpartum issues.

## 3 DISCUSSION

Klippel–Trenaunay Syndrome is a rare congenital vascular and lymphatic disorder affecting 1 in 30 000 to 100 000 individuals.^8^ Although typically benign, it can precipitate thrombotic events, variceal bleeding, and other vascular complications during pregnancy, labor, and the postpartum period. Moreover, KTS‐related symptoms often worsen during pregnancy and the immediate postpartum period, increasing the risk of PE and DVT incidents.^2^, ^6^, ^8^, ^9^, ^10^ As such, in our patient's case, a significant increase in vulvovaginal and left leg vein swelling during her first pregnancy necessitated an investigation for an AVM and a DVT. To reduce the risk of thrombotic events, management during pregnancy includes the use of compression stockings, thrombosis prophylaxis and pain control.^8^

A key consideration in managing pregnant women with KTS is the choice of delivery method, requiring an MDT approach before childbirth.^11^ For this patient, both deliveries were conducted through a caesarean section. Currently, there is a limited record of reported KTS pregnancies. A 2017 literature review reported that 70% of KTS pregnancies opted for caesarean sections, driven by obstetric considerations and the presence of arteriovenous malformations.^11^ Caesarean delivery has also been demonstrated to be preferred in other cases involving vulvovaginal varicosities and pelvic AVMs, further supporting this delivery method as a suitable choice for pregnant patients with KTS.^4^, ^7^, ^11^ However, there have been documented instances of successful vaginal deliveries in similar clinical presentations to this case report. In one such case, labor induction with prostaglandins was used to mitigate vascular risks.^8^, ^11^ The collective review of existing case reports to date prompts consideration that the positive outcomes in our patient might correlate with the absence of significant risk factors identified during screening in both pregnancies. Intriguingly, despite vascular malformations and limb pain in the patient documented by Xiao et al., a vaginal delivery, notably free of complications, was conducted at the patient's insistence, despite KTS obstetric contraindications.^5^

Regarding anesthesia, in cases where neuraxial anesthesia is chosen, it is advisable to undergo an MRI to assess spinal cord tissue, detect AVMs, and mitigate the risk of vascular injury.^8^, ^12^, ^13^ Additionally, in line with prior literature recommendations, we advocate for the use of LMWH during the antenatal and postnatal periods to mitigate thrombotic risks.^2^ This approach outweighs the potential bleeding risks in these patients. Nonetheless, no established thrombosis prevention therapy is outlined in the official management guidelines for this condition.^4^, ^6^, ^8^ Preconception counseling, combined with systematic and patient‐centered care during labor and after childbirth, is essential for decreasing the complication risk.^11^

## 4 CONCLUSION

This case highlights that women with KTS can be safely managed during pregnancy by an MDT approach, involving anesthesiology, hematology, and obstetrics, through careful monitoring and appropriate treatment during the antenatal and postnatal periods. It also suggests that caesarean sections might be the preferred method for childbirth in pregnant women with KTS with concomitant AV malformations and other vascular abnormalities, significantly reducing the risk of thrombotic events. Nevertheless, further research is needed to develop comprehensive obstetric management guidelines for individuals with KTS.

## AUTHOR CONTRIBUTIONS

Inês F. Silva Correia: Substantial contributions to the conception and design of the work, acquisition of patient reports, drafting the manuscript, and providing final approval of the version to be published. Agreed to be accountable for all aspects of the work. Munawar Hussain: Significant support in the acquisition of patient data, critically revising the manuscript, and providing final approval of the version to be published. Agreed to be accountable for all aspects of the work. Jo‐Anne Johnson: Major contributions to the design of the work and interpretation of data, critically revising the manuscript, and providing final approval of the version to be published. Agreed to be accountable for all aspects of the work.

## FUNDING INFORMATION

None.

## CONFLICT OF INTEREST STATEMENT

The authors have no conflicts of interest to declare.

## Data Availability

Data sharing is not applicable to this article as no new data were created or analyzed in this study.
